# Insights into Butyrate Production in a Controlled Fermentation System via Gene Predictions

**DOI:** 10.1128/mSystems.00051-17

**Published:** 2017-07-18

**Authors:** S. Esquivel-Elizondo, Z. E. Ilhan, E. I. Garcia-Peña, R. Krajmalnik-Brown

**Affiliations:** a Biodesign Swette Center for Environmental Biotechnology, Arizona State University, Tempe, Arizona, USA; b Unidad Profesional Interdisciplinaria de Biotecnología, Instituto Politécnico Nacional, Mexico City, Mexico; c School of Sustainable Engineering and the Built Environment, Arizona State University, Tempe, Arizona, USA; Dalhousie University

**Keywords:** butyrate production pathways, PICRUSt, *Prevotellaceae*, hydrogen partial pressure, interconversion reactions, predicted metagenome functional content

## Abstract

Butyrate is a common fatty acid produced in important fermentative systems, such as the human/animal gut and other H_2_ production systems. Despite its importance, there is little information on the partnerships between butyrate producers and other bacteria. The objective of this work was to uncover butyrate-producing microbial communities and possible metabolic routes in a controlled fermentation system aimed at butyrate production. The butyrogenic reactor was operated at 37°C and pH 5.5 with a hydraulic retention time of 31 h and a low hydrogen partial pressure (PH_2_). High-throughput sequencing and metagenome functional prediction from 16S rRNA data showed that butyrate production pathways and microbial communities were different during batch (closed) and continuous-mode operation. *Lactobacillaceae*, *Lachnospiraceae*, and *Enterococcaceae* were the most abundant phylotypes in the closed system without PH_2_ control, whereas *Prevotellaceae*, *Ruminococcaceae*, and *Actinomycetaceae* were the most abundant phylotypes under continuous operation at low PH_2_. Putative butyrate producers identified in our system were from *Prevotellaceae*, *Clostridiaceae*, *Ruminococcaceae*, and *Lactobacillaceae*. Metagenome prediction analysis suggests that nonbutyrogenic microorganisms influenced butyrate production by generating butyrate precursors such as acetate, lactate, and succinate. 16S rRNA gene analysis suggested that, in the reactor, a partnership between identified butyrogenic microorganisms and succinate (i.e., *Actinomycetaceae*), acetate (i.e., *Ruminococcaceae* and *Actinomycetaceae*), and lactate producers (i.e., *Ruminococcaceae* and *Lactobacillaceae*) took place under continuous-flow operation at low PH_2_.

**IMPORTANCE** This study demonstrates how bioinformatics tools, such as metagenome functional prediction from 16S rRNA genes, can help understand biological systems and reveal microbial interactions in controlled systems (e.g., bioreactors). Results obtained from controlled systems are easier to interpret than those from human/animal studies because observed changes may be specifically attributed to the design conditions imposed on the system. Bioinformatics analysis allowed us to identify potential butyrogenic phylotypes and associated butyrate metabolism pathways when we systematically varied the PH_2_ in a carefully controlled fermentation system. Our insights may be adapted to butyrate production studies in biohydrogen systems and gut models, since butyrate is a main product and a crucial fatty acid in human/animal colon health.

## Abstract (precis)

This study demonstrates how bioinformatics tools, such as metagenome functional prediction from 16S rRNA genes, can help understand biological systems and reveal microbial interactions in controlled systems (e.g., bioreactors). Results obtained from controlled systems are easier to interpret than those from human/animal studies because observed changes may be specifically attributed to the design conditions imposed on the system. Bioinformatics analysis allowed us to identify potential butyrogenic phylotypes and associated butyrate metabolism pathways when we systematically varied the PH_2_ in a carefully controlled fermentation system. Our insights may be adapted to butyrate production studies in biohydrogen systems and gut models, since butyrate is a main product and a crucial fatty acid in human/animal colon health.

## INTRODUCTION

Fermentation involves the degradation of organic material by anaerobic microorganisms in an environment with low dissolved oxygen and produces short-chain fatty acids (SCFA) (e.g., butyrate and acetate) and gases, including methane (CH_4_), carbon dioxide (CO_2_), hydrogen sulfide (H_2_S), and hydrogen (H_2_) (1). H_2_ is produced during bacterial fermentation to regulate the electron flow and redox balance (2). H_2_ accumulation is one of the primary causes of low fermentation efficiency (3). Redox reactions [including the oxidation-reduction of ferredoxin and NAD(H)] are the favored mechanisms of H_2_ removal and therefore are crucial for maintaining the fermentation balance (3). Interactions between H_2_ producers and H_2_ consumers (e.g., methanogens, homoacetogens, sulfate reducers) influence the fermentation reactions and therefore the metabolic end products (4). In addition, the hydrogen partial pressure (PH_2_) directly influences the production of fatty acids. Fermentative butyrate and acetate production is favored at low PH_2_s (5) (<0.3 atm), while lactate and propionate are produced at higher PH_2_s (6, 7). Besides depending on the PH_2_, the molar distribution and ratios of fatty acids vary with the type of substrate and the fermentation conditions (8).

Butyrate is a common SCFA produced in important fermentative systems, such as human and animal guts (9), and in microbial butyrate and H_2_ production system (10). Because of this, butyrogenesis (i.e., butyrate production) has been the focus of many gut-related health studies (11, 12) and other biotechnology applications (13–15). Low PH_2_s, acidic to slightly acidic conditions (pHs between 5 and 6.3) (16), and long hydraulic retention times (HRTs) might benefit butyrogenic microorganisms (10). The order *Clostridiales* (in the *Firmicutes* phylum) includes many butyrate producers, such as *Anaerotruncus*, *Faecalibacterium*, *Papillibacter*, *Subdoligranulum*, *Roseburia*, *Butyrivibrio*, *Coprococcus*, *Anaerostipes*, *Clostridium*, *Eubacterium*, and *Shuttleworthia* spp. (9, 17–19). Other butyrogenic microorganisms include *Megasphaera* and *Lactobacillus* spp. in the *Veillonellaceae* and *Lactobacillaceae* families, respectively (20).

**FIG 1:**
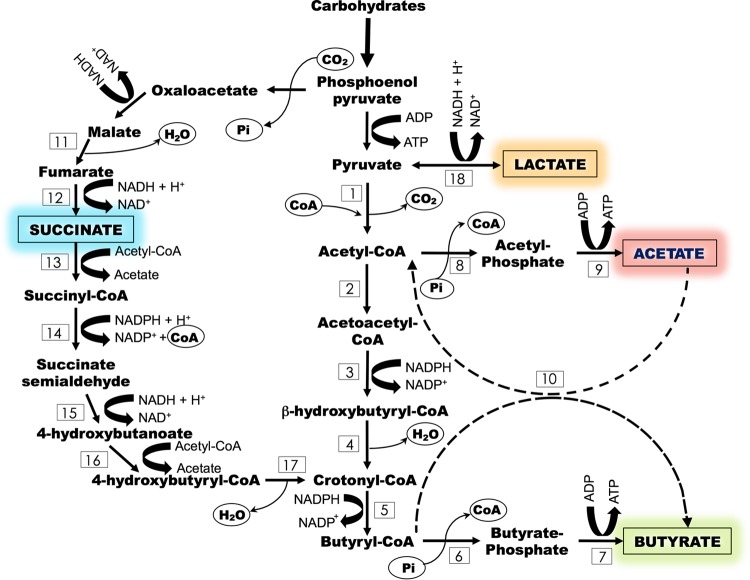
Metabolic routes for butyrate production by (i) direct conversion from carbohydrates via butyrate kinase and (ii) indirect conversion (interconversion reactions) from acetate, succinate, and lactate via butyryl-CoA:acetate-CoA transferase, succinyl-CoA synthetase, and lactate dehydrogenase, respectively, and butyrate kinase. Enzymes: 1, pyruvate-ferredoxin oxidoreductase; 2, acetyl-CoA-acetyltransferase; 3, β-hydroxybutyryl-CoA dehydrogenase; 4, 4-hydroxybutyryl-CoA dehydratase; 5, butyryl-CoA dehydrogenase; 6, phosphotransbutyrylase; 7, butyrate kinase; 8, phosphotransacetylase; 9, acetate kinase; 10, butyryl-CoA:acetate-CoA transferase; 11, malate dehydrogenase; 12, fumarate reductase; 13, succinyl-CoA synthetase; 14, succinate semialdehyde dehydrogenase; 15, 4-hydroxybutyrate dehydrogenase; 16, 4-hydroxybutyrate CoA transferase; 17, 4-hydroxybutyryl-CoA dehydratase; 18, lactate dehydrogenase.

Direct butyrate production from glucose generates 2 mol of H_2_/mol of butyrate produced in accordance with the equation C_6_H_12_O_6_ → butyric acid + 2H_2_ + 2CO_2_ (21). Known microbial butyrate production pathways are summarized in Fig. 1. Butyrogenesis proceeds through butyryl-coenzyme A (butyryl-CoA) generation from acetoacetyl-CoA via the intermediates β-hydroxybutyryl-CoA and crotonyl-CoA. Butyryl-CoA can then be converted to butyrate via two pathways. One involves the generation of butyrate-phosphate via phosphotransbutyrylase, which is then converted to butyrate via butyrate kinase. The second pathway does not involve an intermediate but requires the simultaneous conversion of external acetate to acetyl-CoA and proceeds via butyryl-CoA:acetate-CoA transferase. As noted, the second pathway requires acetate in the medium and is therefore an interconversion reaction. Other common interconversion butyrogenic reactions are succinate and lactate conversion to butyrate (22, 23) (Fig. 1). Succinate conversion to butyrate involves the generation of the butyrate precursor crotonyl-CoA via several intermediates (succinyl-CoA, succinate semialdehyde, 4-hydroxybutanoate, and 4-hydroxybutyryl-CoA), and each step is catalyzed by a specific enzyme (24). Lactate conversion to butyrate proceeds through the generation of pyruvate and thereafter via either butyrate kinase or butyryl-CoA:acetate-CoA transferase (22).

Although these butyrate-producing pathways are well described in the literature, the microbes responsible for direct and indirect butyrate production and the partnerships between butyrogenic and other anaerobic microorganisms have not been clearly identified. Studies that combine the use of controlled systems with deep sequencing techniques and bioinformatics show promise for finding answers to these questions and understanding other microbial interactions that benefit human/animal health and our society. In this study, we used high-throughput 16S rRNA gene sequencing techniques in combination with chemical analysis to study butyrate-producing routes in a bioreactor operated at 37°C and 5.5 pH with a 31-h HRT and a low PH_2_, environmental conditions that would presumably enhance butyrate production. The objectives of this study were (i) to produce butyrate as the main metabolic end product, (ii) to identify potential butyrogenic microorganisms via high-throughput sequencing, and (iii) to understand important microbial partnerships for butyrate production via metagenome functional prediction from marker gene software.

## RESULTS AND DISCUSSION

### Butyrate was the dominant fatty acid produced in the batch and continuous operation modes.

**FIG 2:**
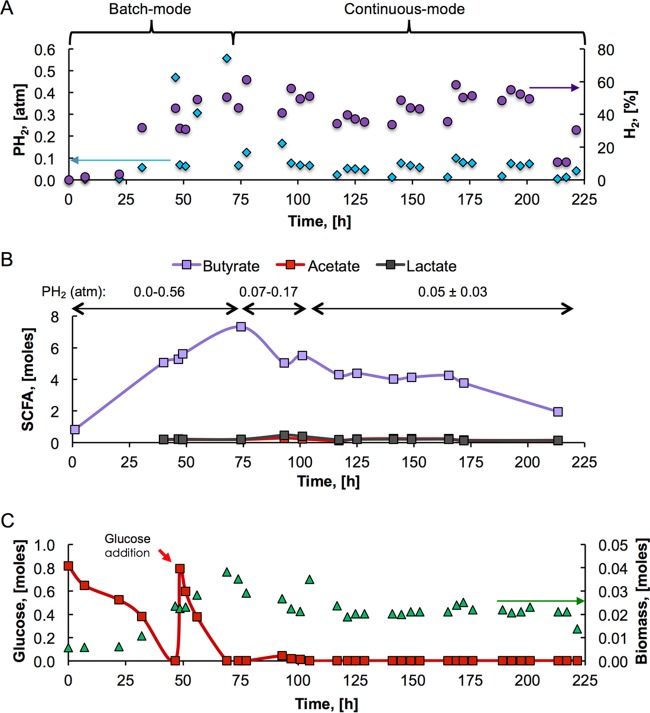
End products of fermentation. (A) PH_2_s (in atmospheres) and H_2_ concentrations (percentages of the biogas) in the gas phase. (B) Butyrate, acetate, and lactate production. (C) Glucose and biomass measured throughout fermentation.

A controlled fermentation system operated at 37°C and a pH of 5.5 with an HRT of 31 h and a PH_2_ of 0.0 to 0.56 atm and inoculated with anaerobic sludge was used to identify butyrate-producing microbial communities and possible metabolic routes for butyrate production. Results presented in Fig. 2 indicate that the environmental conditions selected and the low-to-moderate PH_2_ (0 to 0.56 atm, 0 to 0.41 mM) during batch mode operation and the low PH_2_ (0.05 ± 0.03 atm, 0.02 to 0.06 mM) during continuous-mode operation lead to continuously high butyrate production.

As shown in Fig. 2A, H_2_ accumulated in the closed system (batch-mode operation), and after a second addition of glucose during h 49 (Fig. 2C), the PH_2_ reached 0.56 atm (0.41 mM). During continuous operation, the PH_2_ was mechanically controlled through biogas venting. After completion of the first cycle of continuous operation with an HRT of 31 h, the PH_2_ was kept at 0.05 ± 0.03 atm (0.02 to 0.06 mM) for 4 cycles of 31 h each. The low PH_2_ limited the growth of hydrogenotrophic microorganisms such as methanogens and acetogens. Quantitative PCR (qPCR) analysis of archaeal 16S rRNA and the *fthfs* acetogen marker gene confirmed the absence of methanogenic and acetogenic activity during continuous operation. Copies of the archaeal 16S rRNA gene were below the limit of detection in the batch system and during all cycles of continuous-mode operation. The *fthfs* acetogen marker gene was only detected in the sample collected during batch mode operation (3.07 × 10^4^ copies ml^−1^), when the PH_2_ was not controlled.

As depicted in Fig. 2B, butyrate was the main fatty acid produced during fermentation. The butyrate concentrations in the batch and continuous operation modes were 84 mM (7.3 mol) and up to 63 mM (5.5 mol), respectively. The concentration of butyrate was significantly correlated with the concentration of H_2_ in the biogas produced (Pearson’s *R* [correlation coefficient] of 0.81, significant at the 0.01 level). This positive correlation is in accordance with the equation above; H_2_ was produced from glucose along with butyrate. No other significant parametric correlation between butyrate or H_2_ and other variables of the process was observed. Besides butyrate, acetate and lactate were the other main fatty acids detected during fermentation (Fig. 2B). The concentrations of acetate (2.2 to 4.7 mM, 0.1 to 0.3 mol) and lactate (1.7 to 5.2 mM, 0.15 to 0.5 mol) were low compared to the butyrate concentrations. While acetate is a source of carbon and energy for many metabolic reactions (25), lactate is an intermediate of fermentation reactions that produce acetate, butyrate, and propionate (22, 26).

**FIG 3:**
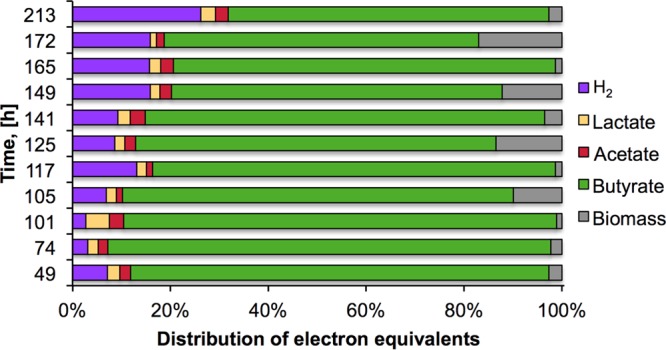
Electron equivalent distribution from glucose to measured products.

Figure 2C shows that during batch mode operation, glucose was completely consumed and up to 0.38 mol of biomass was accumulated (2.5 mmol of biomass liter^−1^). In continuous-mode operation with a controlled low PH_2_, most of the glucose (>95%) continuously supplied at 10 g·liter^−1^ was consumed and biomass accumulation was kept close to 0.02 mol (1.5 mmol of biomass liter^−1^). A constant biomass concentration confirms the stable operation of the bioreactor. According to the electron distribution analysis presented in Fig. 3, butyrate, acetate, lactate, H_2_, and biomass were the main end products of fermentation; accounting for >85% of the electrons from the total glucose added during 221 h of operation. The remaining ~15% of electrons were possibly distributed among other products, such as alcohols and other fatty acids. However, the electron balance suggests that the concentrations of other end products were low because of low production or their consumption in interconversion reactions.

### *Prevotellaceae*, *Actinomycetaceae*, and *Ruminococcaceae* were highly abundant at low PH_2_ and during high butyrate production.

**FIG 4:**
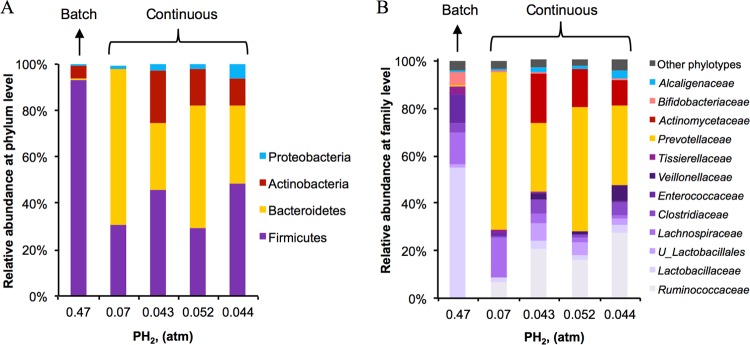
Relative abundance of microorganisms identified at the phylum (A) and family (B) levels throughout fermentation at different PH_2_s. The sample from the batch system was collected after 47 h of fermentation. The four samples from the continuous system were collected at 74, 145, 149, and 173 h, respectively. *U*_ indicates an unidentified microorganism within the taxonomic classification.

To identify key bacteria and important interactions involved in the butyrogenic reactor, we used DNA-based techniques to analyze the structure of the microbial community and its changes throughout fermentation. As expected, the microbial communities that developed in the reactor were different between the closed (batch) and continuous systems. These differences can be clearly seen in Fig. 4. During batch operation, there was no external control of PH_2_; thus, the mode of operation and PH_2_ caused batch microbial communities to diverge from continuous communities.

Figure 4 shows the distribution of microbial phylotypes at the phylum and family levels at different time points throughout the process, corresponding to different PH_2_s. In the sample corresponding to the batch system (at a PH_2_ of 0.47 atm), 93% of the phylotypes belong to the *Firmicutes* phylum; the phyla *Actinobacteria*, *Bacteroides*, and *Proteobacteria* were also present but at minor relative abundances. The most abundant phylotypes at the family level in this closed system were *Lactobacillaceae*, *Lachnospiraceae*, and *Enterococcaceae* in the phylum *Firmicutes*, followed by *Bifidobacteriaceae* (*Actinobacteria*), *Tissierellaceae* (*Firmicutes*), and *Clostridiaceae* (*Firmicutes*), to a lesser extent.

After reactor operation was switched to continuous mode and a lower PH_2_ was maintained (0.02 to 0.17 atm), the microbiota structure changed drastically, yielding a higher distribution of *Bacteroidetes*, *Actinobacteria*, and *Proteobacteria* phylotypes. At the beginning of the first cycle of continuous-mode operation (PH_2_ of 0.07 atm), phylotypes in the *Bacteroidetes* phylum reached their highest relative abundance (67%), followed by phylotypes of the *Firmicutes* phylum (31%). After the PH_2_ was further decreased to ~0.05 atm (~0.03 mM) by the beginning of the second cycle, the relative abundance of *Bacteroidetes* decreased to 29%, whereas the abundance of *Firmicutes* and *Actinobacteria* increased to 46 and 23%, respectively. The microbial populations represented by these phylotypes remained at a relatively constant abundance for the rest of the continuous operation at a PH_2_ of 0.043 to 0.052 atm (0.05 ± 0.005 atm), 29 to 49% *Firmicutes*, 29 to 52% *Bacteroidetes*, 12 to 23% *Actinobacteria*, and 1.5 to 6% *Proteobacteria*.

The most abundant phylotypes at the family level identified under continuous operation at a low PH_2_ were *Prevotellaceae*, *Ruminococcaceae*, and *Actinomycetaceae*, followed by *Lachnospiraceae*, *Clostridiaceae*, and unidentified *Lactobacillales*, to a lesser extent. Previously described butyrate-producing microorganisms are in the order *Clostridiales* (*Firmicutes*) within four families, *Ruminococcaceae*, *Lachnospiraceae*, *Clostridiaceae*, and *Eubacteriaceae* (9, 17, 18). Phylotypes in three of these families (i.e., *Ruminococcaceae*, *Lachnospiraceae*, and *Clostridiaceae*) were detected at considerable abundance during batch and continuous operation. Although not all of the genera within these families are butyrate producers (19), the high relative abundance of these families, along with high butyrate production, suggests that microorganisms belonging to at least some of these families were responsible for the high butyrate production observed throughout fermentation. Moreover, *Prevotellaceae* is also made up of butyrate producers, including *Prevotella ruminicola*, which generates butyrate, in addition to other SCFAs, from pectin (27). Possible metabolic routes that lead to butyrate production (Fig. 1) in the butyrogenic reactor and possible associations of these routes with identified phylotypes (Fig. 4) were investigated via PICRUSt (phylogenetic investigation of communities by reconstruction of unobserved states) (28), and the results are discussed in the following section.

### *Prevotellaceae*, *Clostridiaceae*, and *Lactobacillaceae* were potential butyrate producers in the bioreactor.

**FIG 5:**
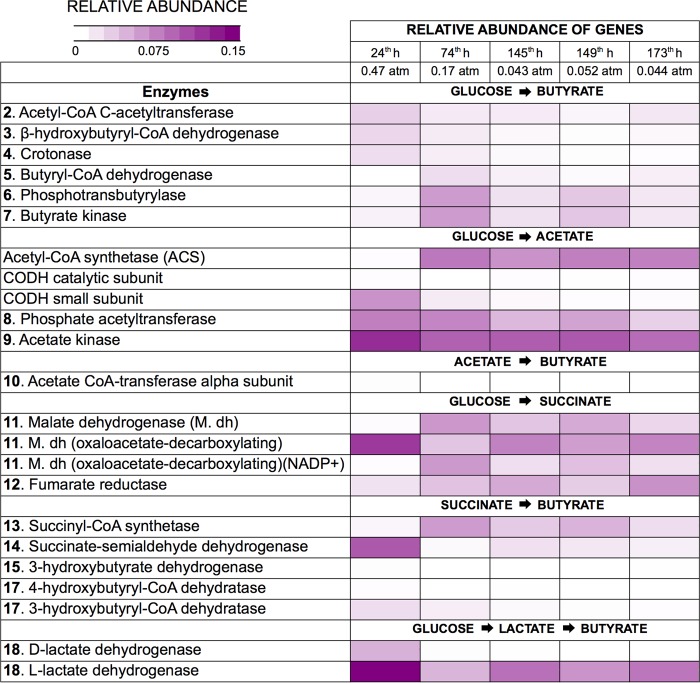
Relative abundances (percent) of genes that code for key enzymes involved in direct and indirect butyrate production during glucose fermentation at different PH_2_s. Gene abundances were predicted with PICRUSt. The range from purple to white corresponds to higher to lower relative abundances. The numbering corresponds to that in Fig. 1.

The predicted relative abundance of key metabolic genes involved in butyrate production and the metagenome contribution of identified phylotypes to the relative abundance of these genes were predicted from community structure (16S rRNA gene) data by using PICRUSt. Because butyrate can be directly produced from glucose and indirectly derived from interconversion reactions (12), we focused on the presence and relative abundance of genes that code for enzymes involved in butyrate production from glucose, acetate, succinate, and lactate. The predicted relative abundances of these genes and their predicted metagenome contributions are summarized in Fig. 5 and Table 1, respectively.

**TABLE 1:** Predicted metagenome contributions of phylotypes (at the family level) to genes encoding key enzymes involved in the production and consumption of butyrate, acetate, succinate, and lactate

Enzyme (no.) and phylotype	Contribution of phylotype (%)^a^	
	Batch	Continuous
4-Hydroxybutyryl-CoA dehydratase (17)		
*Ruminococcaceae*	0	95 ± 5
*Clostridiaceae*	100	3 ± 1
Butyrate kinase (7)		
*Prevotellaceae*	15	92 ± 7
*Ruminococcaceae*	0	3 ± 0
*Clostridiaceae*	44	0
*Lachnospiraceae*	1	1 ± 0
*Lactobacillaceae*	35	0
Acetate kinase (9)		
*Prevotellaceae*	16	34 ± 21
*Ruminococcaceae*	0	43 ± 18
*Clostridiaceae*	57	1 ± 1
*Lachnospiraceae*	12	7 ± 6
*Actinomycetaceae*	0	12 ± 4
Fumarate reductase (12)		
*Clostridiaceae*	12	1 ± 1
*Lactobacillaceae*	25	2 ± 1
*Alcaligenaceae*	51	96 ± 2
Succinyl-CoA synthetase (13)		
*Prevotellaceae*	20	61 ± 16
*Actinomycetaceae*	0	20 ± 17
*Bifidobacteriaceae*	67	1 ± 0
Lactate dehydrogenase (18)		
*Ruminococcaceae*	0	57 ± 1
*Lactobacillaceae*	67	6 ± 1
*Actinomycetaceae*	0	7 ± 6

a Average of three samples ± standard deviation.

The predicted relative abundance of the gene encoding acetate kinase (enzyme no. 9), a key enzyme involved in acetate production and consumption, was relatively high (0.12%) during operation in batch mode (h 47) and continuous mode (0.09% ± 0.004%) (Fig. 5) compared to the relative abundance of almost 7,000 genes/enzymes identified in the analysis (see Data Set S1 in the supplemental material). Phosphate acetyltransferase, a key enzyme (no. 8) involved in acetate production and consumption, was also abundant throughout fermentation (Fig. 5). Low acetate concentrations during fermentation (2.2 to 4.7 mM) suggest the consumption of acetate for several purposes, including energy generation and the production of other fatty acids. On the basis of their predicted contribution to the presence of these genes, the main phylotypes involved in acetate production and/or consumption were *Prevotellaceae*, *Clostridiaceae*, and *Lachnospiraceae* during batch mode operation and *Prevotellaceae*, *Ruminococcaceae*, and *Actinomycetaceae* during continuous-mode operation (Table 1). The acetate interconversion reaction to butyrate was possibly not the main butyrate production route, since the gene that codes for butyryl-CoA:acetate CoA-transferase, the enzyme (no. 10) that catalyzes the conversion of acetate to butyrate, was predicted to exist at a low relative abundance (≤0.002%) in both the batch and continuous modes (Fig. 5).

10.1128/mSystems.00051-17.1 DATA SET S1  PICRUSt metagenome predictions and metagenome contributions. Download DATA SET S1, XLSX file, 0.4 MB. Copyright © 2017 Esquivel-Elizondo et al. 2017 Esquivel-Elizondo et al. This content is distributed under the terms of the Creative Commons Attribution 4.0 International license.

The fermentation process aimed at butyrate production was enriched in genes encoding enzymes involved in lactate generation from pyruvate (lactate dehydrogenase [no. 18]) (Fig. 4). Lactate dehydrogenase is involved in lactate fermentation to butyrate and other acids. Similar to acetate, the low concentration (1.7 to 5.2 mM) of lactate measured throughout fermentation suggests its possible consumption through several interconversion reactions. The main phylotype predicted to contribute to the presence of lactate dehydrogenase was *Lactobacillaceae* during batch mode operation and *Ruminococcaceae* during continuous-mode operation (Table 1).

Genes encoding enzymes involved in butyrate production were highly abundant throughout fermentation (Fig. 5). The main phylotypes that contributed to the presence of butyrate kinase, the enzyme that catalyzes the conversion of butyrate phosphate to butyrate (Fig. 1, enzyme no. 7), were *Clostridiaceae*, *Lactobacillaceae*, and *Prevotellaceae* during batch mode operation and *Prevotellaceae* during continuous-mode fermentation. *Lactobacillaceae* most likely produced butyrate and lactate in the batch and continuous modes, respectively, since, as already mentioned, phylotypes related to this family also considerably contributed to the genes involved in lactate production and consumption. In addition, on the basis of the high butyrate and low lactate concentrations measured in the bioreactor, it is possible that *Lactobacillaceae* produced butyrate via the lactate interconversion reaction. *Prevotellaceae* and *Clostridiaceae*, on the other hand, were not involved in lactate production/consumption. Hence, these two phylotypes potentially produced butyrate directly from glucose and/or via the succinate interconversion reaction.

**FIG 6:**
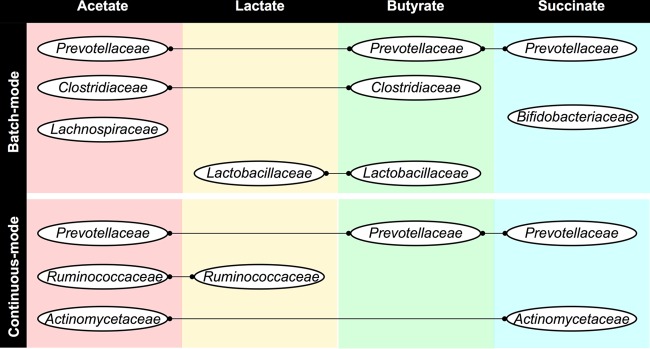
Phylotypes (at the family level) predicted to contribute to genes that code for key enzymes (summarized in Table 1) involved in the production and consumption of acetate, lactate, butyrate, and succinate.

*Prevotellaceae* and *Actinomycetaceae* are comprised of succinate producers and consumers (29–31). Accordingly, these phylotypes were the main contributors to succinate-consuming enzymes (i.e., succinyl-CoA synthetase [no. 13] and succinate/semialdehyde dehydrogenase [no. 14]) in the butyrogenic reactor operated in continuous mode. On the basis of its important contribution to the genes that encode butyrate kinase and succinyl-CoA synthetase, it is possible that microorganisms in the family *Prevotellaceae* produced butyrate via succinate. Members of the family *Actinomycetaceae* produced/consumed acetate and succinate, but according to the metagenome prediction, they were not directly involved in butyrate production. *Clostridiaceae* did not contribute to the presence of succinate consumption (enzymes no. 13 and 14 in Fig. 1) but possibly produced butyrate directly from glucose. Figure 6 summarizes the main phylotypes predicted to contribute to acetate, lactate, butyrate, and succinate production and/or consumption during the two stages of fermentation, the batch and continuous modes of operation.

Besides conversion to butyrate, succinate can also be converted to propionate via methylmalonyl-CoA and propionyl CoA:succinate CoA transferase (32). Nonetheless, the predicted abundance of the genes that code for these enzymes was close to zero throughout fermentation. Hence, on the basis of metabolic predictions, succinate conversion to propionate was not an important metabolic reaction in the bioreactor.

### Phylotypes in the families *Actinomycetaceae*, *Ruminococcaceae*, and *Lachnospiraceae* and other fermenters enhanced butyrate production via the generation of butyrate precursors.

Besides butyrate producers, phylotypes most closely related to known lactate, succinate, and acetate producers within the *Firmicutes* and *Actinobacteria* phyla were dominant in the butyrogenic reactor in continuous operation and at a low PH_2_ (Fig. 4). The family *Ruminococcaceae* (*Firmicutes*) is composed of acetate, lactate, and butyrate producers (18). Metagenome prediction analysis suggests that, in the bioreactor, microorganisms in the family *Ruminococcaceae* were mainly involved in acetate and lactate production/consumption but not directly involved in butyrate production (Table 1), as expected. Members of the family *Actinomycetaceae* (*Actinobacteria*) are known lactate, acetate, and succinate producers (31), and they were predicted to contribute greatly to the abundance of genes involved in acetate and succinate metabolism. Members of the families *Lactobacillaceae* and *Veillonellaceae* (*Firmicutes*), also detected at high relative abundance (Fig. 4), produce succinate, lactate, acetate, and butyrate from organic substrates (20). Other fermenters identified in the process include microorganisms in the families *Lachnospiraceae* (33) and *Bifidobacteriaceae* (34, 35) (Fig. 4). *Bifidobacterium* spp. (*Actinobacteria*) are known to promote butyrate production via cross-feeding with butyrogenic microorganisms (36). The identification of numerous SCFA producers and butyrogenic microorganisms, along with the detection of high butyrate concentrations, compared to other SCFAs during fermentation, suggests that, in the bioreactor, butyrate was produced via interconversion reactions that involved more than one group of microbes in addition to its direct conversion from glucose. Metagenome prediction analysis (presented in Fig. 5 and Table 1 and summarized in Fig. 6) suggests that members of the families *Ruminococcaceae*, *Actinomycetaceae*, and *Lachnospiraceae* and other identified SCFA producers indirectly contributed to butyrate production via the generation of acetate, lactate, and succinate for interconversion reactions.

In this study, high-throughput sequencing in combination with chemical analyses allowed us to better understand intermicrobial conversions that were taking place in a closed and controlled fermentation system. Our results have implications for systems where butyrate production is important, such as the human colon. Butyrate is the preferred energy source of colonic epithelial cells; it is a regulator of mucosal gene expression, differentiation, and apoptosis, and its anti-inflammatory properties help prevent/treat colonic mucosa diseases such as ulcerative colitis and colorectal cancer (9, 37). Despite its importance, there is little information on the distribution and abundance of butyrate-producing enzymes in gut bacteria. This controlled bioreactor study provides insights into complex interactions among microorganisms that are involved in butyrate production. Interestingly, phylotypes identified in our bioreactor, including *Prevotellaceae*, *Ruminococcaceae*, *Lachnospiraceae*, and *Bifidobacteriaceae*, are most closely related to bacteria commonly found in the human colon and the mammalian gastrointestinal tract (19, 38).

### Conclusions.

High-throughput 16S rRNA gene sequencing analysis revealed that both the PH_2_ and the type of operating condition (batch or continuous mode) had an important influence on the microbial community structure and function, specifically for microbes involved in butyrate production. Predicted metagenome functional genes involved in butyrate production, as well as acetate, lactate, and succinate production and consumption, suggest that butyrate was indirectly produced from glucose fermentation intermediates (succinate, lactate, and possibly acetate) and through interconversion reactions, in addition to being produced directly from glucose. Butyrate producers and main butyrate production routes in the bioreactor changed during fermentation according to the environmental conditions (i.e., batch versus continuous-mode operation and PH_2_). Direct production from glucose and lactate interconversion reactions was dominant in the batch system, while succinate interconversion reactions were the main butyrate production route in the continuous system at a low PH_2_ (0.05 ± 0.03 atm). The potential phylotypes involved in butyrate production from glucose, succinate, and lactate were *Clostridiaceae*, *Prevotellaceae*, and *Lactobacillaceae*, respectively. *Prevotellaceae*, *Lactobacillaceae*, and other potential butyrate producers (e.g., *Veillonellaceae*) most likely partnered with succinate, lactate, and acetate producers in the *Actinomycetaceae*, *Ruminococcaceae*, *Lachnospiraceae*, and other fermentative microbial families for butyrate production. Identifying all potential microbial butyrate production routes and contributors is essential for the optimization of systems where butyrate is the desired end product.

## MATERIALS AND METHODS

### Inoculum and bioreactor setup.

The inoculum was anaerobic sludge from a bench scale anaerobic digester initially inoculated with cow manure and periodically fed ground fruit and vegetable scraps (39). To reduce the effect of microbial H_2_ consumers, the inoculum was heat pretreated at 80°C for 35 min to eliminate methanogenic activity. Homoacetogenic activity was limited by a low PH_2_. To ensure a controlled system, biological H_2_ removal was mimicked by mechanically venting the biogas that was generated.

Fermentation was carried out in a 30-liter continuous-flow stirred tank reactor with a 15-liter liquid volume and a 10% (vol/vol) inoculum (30 g of volatile suspended solids liter^−1^) with a medium consisting of glucose (10 g·liter^−1^) and a previously reported mineral solution (40). The reactor was operated at 37°C, the pH was maintained at 5.5, and the liquid phase was stirred at 150 rpm. Before operation, anaerobic conditions were established by sparging the headspace of the reactor with a current of N_2_ for 30 min. The fermentation was initialized as a batch mode operation (closed system) to accumulate biomass. After 48 h of fermentation, the accumulated biogas was completely released (until a headspace gauge pressure of 0 atm was reached) and more glucose (10 g·liter^−1^) was added to increase the active biomass. Biogas was again completely released before a transition to continuous-mode operation at 74 h. During this stage, glucose was continuously fed (8.1 ± 0.05 ml·min^−1^) to the bioreactor at a concentration of 10 g·liter^−1^. The continuous-mode system consisted of five cycles, each one with an HRT of 31 h.

During the first cycle (the first 31 h) of continuous operation (74 to 105 h), the reactor gauge pressure was kept between 0.13 and 0.41 atm by intermittent biogas release. From 105 h on (the second cycle), the biogas was continuously and constantly vented through a valve located at the top of the reactor to maintain the operation pressure at 0.136 atm. The purpose of constantly releasing the biogas was to enhance butyrate and fermentative acetate production by maintaining a low PH_2_.

PH_2_s were determined from the total gauge pressure in the reactor headspace and Dalton’s law of partial pressures, PH_2_ = (% H_2_/100) × total pressure (atm). H_2_ concentrations in the liquid phase were estimated with Henry’s law by using the H_2_ partial pressure and the equilibrium constant for H_2_ and water at 37°C (1,341 atm·liter·mol^−1^) (41).

### Analytical methods.

The H_2_ in the headspace was periodically measured with a gas-tight syringe (0.15-ml injection volume) and a gas chromatograph (Gow-Mac 580) equipped with a thermal conductivity detector and a silica gel 60/80 column (18 feet by 1/8 in. by 0.085 in.; Alltech) under previously reported conditions (40). To determine H_2_ concentrations, a calibration curve was made at 2 lb/in^2^ (0.136 atm). When the system pressure was >2 lb/in^2^, biogas release was required prior to gas sample analysis.

The glucose concentration in liquid samples was quantified by the 3,5-dinitrosalicylic acid colorimetric method as previously reported (40). The biomass concentration was estimated by quantifying protein by the Bradford method and by assuming that protein constituted 25% of the dry biomass weight (42).

Fatty acid composition was determined with a high-performance liquid chromatography (HPLC) system and liquid samples (30-μl injection volume) periodically taken from the bioreactor. The HPLC system (PerkinElmer 200) was equipped with a UV detector (measurements at 210 nm) and a Prevail Organic column (150 by 4.6 nm; Grace) with a KH_2_PO_4_ (25 mM, 2.5 pH) mobile phase (1 ml·min^−1^) as previously reported (40).

### Molecular microbial ecology analysis. (i) DNA extraction.

Five samples were taken at different operation times, (i) during batch mode operation at 47 h at a PH_2_ of 0.47 atm, (ii) at the beginning of continuous operation (74 h) at a PH_2_ of 0.07 atm, (iii) at 145 h during the third cycle at a PH_2_ of 0.043 atm, (iv) at 149 h at a PH_2_ of 0.052 atm, and (v) at 173 h during the fourth cycle at a PH_2_ of 0.044 atm.

For each of the five samples, cells were harvested from 1.5 ml of broth in a sterile tube by centrifugation at 14,000 rpm for 5 min, followed by decantation of the supernatant. The pellet was resuspended in 0.5 ml of extraction buffer. DNA was extracted by the cetyltrimethylammonium bromide protocol (43). The purified DNA was eluted with 40 µl of Milli-Q water and kept at −20°C before pyrosequencing and qPCR. The DNA concentrations and purity of each sample were determined by measuring absorbance at wavelengths of 260 and 280 nm with a NanoDrop spectrophotometer (NanoDrop Technology, Rockland, DE).

### (ii) High-throughput sequencing and bioinformatic and statistical analyses.

Bacterial tag-encoded FLX amplicon pyrosequencing was performed at the Research and Testing Laboratory (Lubbock, TX). Bacterial primers 104F and 530R were used to amplify the V2 and V3 hypervariable regions of the 16S rRNA gene spanning nucleotides 137 to 242 and 433 to 497, respectively (numbering based on the *Escherichia coli* 16S rRNA gene) (44). Amplicons were sequenced with the FLX-Titanium System Genome Sequencer. A total of 30,116 raw sequence reads were received.

The QIIME 1.8.0 pipeline was used to process raw sequences (45). Sequences with at least one of the following characteristics were omitted from downstream analysis: a length of <200 bp, a quality score of ≤25, any primer or barcode mismatches, and more than six homopolymers. From the sequences that passed the quality filtering, operational taxonomic units (OTUs) were picked on the basis of 99% sequence similarity by using the UCLUST algorithm (46). The most abundant sequence of each cluster was picked as the representative sequence. Taxonomy was assigned to the representative sequences by comparing them to the Greengenes database (47). Representative sequences were aligned with PyNAST (48). Chimeras within the representative and aligned sequences were identified and removed with Chimera Slayer (49). A BIOM-formatted OTU table (Data Set S2) was constructed from the representative sequences by excluding chimeras and singletons. To avoid biases that occur when sampling various species in a community, the OTU table was subsampled (rarefied) at 4,385 sequences with NumPy, a pseudorandom number generator (50). The final total number of sequences was 28,514 (7,840, 7,032, 4,720, 4,597, and 4,385 for each sample, respectively), and the final OTU number was 3,616.

10.1128/mSystems.00051-17.2 DATA SET S2  OTU table constructed from the representative sequences, excluding chimeras and singletons. Download DATA SET S2, TXT file, 0.1 MB. Copyright © 2017 Esquivel-Elizondo et al. 2017 Esquivel-Elizondo et al. This content is distributed under the terms of the Creative Commons Attribution 4.0 International license.

The bioinformatics tool PICRUSt (28), was used to predict metagenomes from the 16S rRNA reads. For this, closed reference OTUs were picked with QIIME 1.8.0 (45). Pearson’s parametric correlation was performed with the Statistical Package for the Social Sciences (SPSS) software. A *P* value of <0.05 was accepted as significant.

### (iii) Real-time qPCR.

Extracted DNA was normalized to 10 ng·µl^−1^ so that the different samples were equivalent for comparison. Real-time qPCR based on SYBR green technology (TaKaRa) was used to confirm the absence of hydrogen consumers by targeting the 16S rRNA gene in general archaea (51) and the functional gene for formyl tetrahydrofolate synthetase (*fthfs*) in homoacetogenic bacteria (52). The primers, probes, and plasmid DNA standards used in this study were previously reported (51, 52). Each assay was performed in triplicate by using a six-point standard curve along with the samples. The concentrations of the primers, probes, and reagents used and the amplification conditions used were those previously described for archaea and *fthfs* (53). The samples were amplified in an Eppendorf RealPlex 4S thermocycler (Eppendorf, Germany), and fluorescent signal data were processed with LightCycler software.

### Electron balance.

The electron balance helps understand the percentage distribution of electrons provided in substrates (i.e., glucose) to the end products identified (i.e., butyrate, acetate, lactate, biomass, and H_2_) (54). For this analysis, the numbers of moles of glucose and end products measured at different time points throughout fermentation were converted to electron equivalents. The numbers of electron equivalents per mole were as follows: glucose, 24; H_2_, 2; lactate, 12; acetate, 8; butyrate, 20; biomass (considering ammonia as the nitrogen source), 20 (54). The distribution of electron equivalents from substrates to end products was calculated by dividing the number of electron equivalents of each end product by the number of electron equivalents provided as the substrate and multiplying the result by 100.

### Accession number(s).

Sequences were submitted to the NCBI Sequence Read Archive and assigned accession numbers SAMN02440024 to SAMN02440028.
